# How Does the Built Environment in Compact Metropolitan Cities Affect Health? A Systematic Review of Korean Studies

**DOI:** 10.3390/ijerph16162921

**Published:** 2019-08-14

**Authors:** Dong Ha Kim, Seunghyun Yoo

**Affiliations:** 1 Department of Public Health Sciences, Graduate School of Public Health, Seoul National University, Seoul 08826, Korea; 2 Institute of Health and Environment, Seoul National University, Seoul 08826, Korea

**Keywords:** built environment, health promotion, compact city, metropolitan scale, systematic review, Korea

## Abstract

This systematic review aimed to examine the associations between health-related outcomes and the built environment (BE) characteristics of compact metropolitan cities in Korea using the Preferred Reporting Items for Systematic Reviews and Meta-Analyses (PRISMA) framework. Searching the three Korean academic databases and PubMed, two independent reviewers identified 27 empirical articles published between 2011 and 2016. Data extracted for review included the study characteristics, the variables and measurement methods related to the BE and health-related outcomes, and the findings on the associations between the BE characteristics and health-related outcomes. Vote counting was used to assess the consistency of associations and the direction of associations between the BE characteristics and health-related outcomes. All of the reviewed studies used cross-sectional designs. The objective BE qualities were commonly examined. The BE characteristics associated with health-related outcomes in the reviewed articles included land use, street environment, transportation infrastructure, green and open spaces, and neighborhood facilities. Street environment, transportation infrastructure, and green and open spaces had consistent positive associations with physical health. Mixed land use and neighborhood facilities, however, had inconsistent associations with physical health. Generally, insufficient findings were reported in the association between the BE characteristics and mental and social health. The accessibility of the BE in a compact urban environment was the prominent attribute related to health promotion, health challenges, and health equity. An international comparative analysis of compact cities with different urban contexts and scale is required. Interdisciplinary urban health strategies are recommended based on the associations between the BE characteristics and health-related outcomes.

## 1. Introduction

As health promotion strategies targeting policy, system, and environment changes become more important, the built environment (BE) is becoming a key element of interventions [1]. The BE encompasses daily living environments created by people, including residential environments, transportation systems, food-related infrastructure, neighborhood parks, and green spaces [2]. The BE is familiar and relevant to urban residents and can be an environmental determinant of health.

In the Global Report on Urban Health, the World Health Organization (WHO) indicated the influence BE conditions have on public health, notably in the urban context [3]. This is because the BE is directly or indirectly related to urban problems, such as urban sprawl, urban crime, solid waste, energy overconsumption, and climate change, affecting both urban and non-urban residents’ health [4,5]. To create a healthy and salutogenic city, urban design strategies, advocacy, and policies should focus on the relationship between the BE and health within the urban context [5].

The impact of BE on public health is a topic that has been studied extensively around the world. Existing literature suggests that mixed land use, street connectivity, public transportation, and green space are positively related to physical activity and weight loss [6,7]. Furthermore, increased accessibility to public transport and neighborhood walkability contributed to reduced risk of depression and dementia [8,9]. A systematic review reported that the accessibility and diversity of destinations were associated with social cohesion [10]. However, there was variation in the size and form of the cities where these studies were conducted; few studies have included an urban context of a metropolitan scale. This reflects a research gap in *what* aspects of and *how* the BE in metropolitan cities affects public health. 

The Organisation for Economic Co-operation and Development (OECD) recommends that metropolitan cities adopt a compact urban design to solve urban problems caused by high population density and inefficient land use [11]. Metropolitan scale cities in South Korea (Korea hereafter), which has high urban compactness among OECD countries, are the capital city (Seoul) and six metropolitan cities (Busan, Incheon, Daegu, Daejeon, Gwangju, and Ulsan). These cities have: (1) Population density in urban land >1000 pop/km^2^, (2) an independent budget management system, (3) public transit infrastructure (subway and bus), and (4) an urban master plan for compact urban design [12]. According to this plan, these cities have established high-density development strategies and promoted multi-land use around public transport facilities.

The health status of residents in Korean metropolitan cities is complicated. The following indicators were better than the WHO average in 2016: Life expectancy at birth was 82.7 years, prevalence of insufficient physical activity was 35.4%, prevalence of depressive disorders was 3.0%, and the prevalence of obesity was 26.0% [13,14]. On the other hand, the following indicators were worse than the WHO average in 2016: Suicide rate (per 100,000 population) was 26.1%, alcohol per capita consumption was 10.2 L, and asthma mortality rate (per 100,000 population) was 4.9%. The BE is considered a core determinant of the complex health situation in metropolitan cities [4,5]. 

Although Korean studies on the BE and health have been conducted since 1978, there have been scare attempts to integrate the results of BE characteristics that affect the health of urban populations. These studies have focused on different BE and health-related variables, and have used different methods and measurement criteria [15]. They also lack coherence in spatial units and variable measurement, making it difficult to ascertain definite associations with a single study [16]. To synthesize these results, this systematic review examines the associations between health-related outcomes and the BE characteristics of compact metropolitan cities in Korea.

## 2. Materials and Methods

### 2.1. Search Strategy

This study followed the Preferred Reporting Items for Systematic Reviews and Meta-Analyses (PRISMA) framework for conducting and reporting systematic reviews [17]. The review included literature from 2011 to December 2016, after the establishment of the third Korean National Health Promotion Plan (Health Plan 2011–2020). For the first time, this national plan included strategies to create a healthy environment for public health promotion. Moreover, most of the relevant articles were published during this time period. 

### 2.2. Eligibility

Studies were selected by applying the eligibility criteria of settings, study design, methods, and outcome measures. Inclusion criteria were:

- Studies conducted in Korean metropolitan cities.
- Studies that objectively (e.g., geographic information systems) or subjectively (i.e., survey, scale) measured the BE reported as independent variables.
- Studies that objectively (e.g., medical examination) or subjectively measured health-related outcomes (e.g., symptoms, mortality, physical, mental, and social functioning, perceived health status, health-related behaviors) [18].
- Peer-reviewed and fully published articles.
- Published articles written in Korean or English.

Exclusion criteria were:

- Studies that objectively or subjectively measured indoor facilities and working facilities reported as independent variables.
- Studies that objectively or subjectively measured the natural environment (i.e., non-man-made physical environment) reported as independent variables.
- Systematic reviews.
- Qualitative studies.
- Studies conducted to develop the BE measuring instrument that does not analyze the correlation with health.
- Descriptive studies with only bivariate analysis.

### 2.3. Information Sources and Search Terms

Between February and June 2017, we conducted literature searches in three Korean academic databases (DBpia, KISS, and Riss4U) and PubMed. The search terms were identified from previous related reviews [19] and the following terms were used to search for relevant articles: ‘South Korea’ AND (‘built environment’ or ‘urban environment’ or ‘neighborhood environment’ or ‘physical environment’) AND (‘health’ or ‘physical health’ or ‘mental health’ or ‘social health’ or ‘health-related behavior’ or ‘health promotion’). We excluded urban form from the search because it is a broad concept that includes urban size, density, shape, structure, and configuration of settlements [20].

### 2.4. Data Extraction

Data extracted included the characteristics (i.e., publication year, author disciplines, study location, participants, sample size, sampling method, data source, analysis method), the BE variables and measurement methods, health-related variables and measurement methods, and significant and non-significant findings on the correlation between the BE characteristics and health-related outcomes.

To include all the BE variables across studies, we listed and categorized each variable and construct of the BE characteristics (i.e., land use, street environment, transportation infrastructure, green and open spaces, and neighborhood facility) (Table 1). The BE measurement methods were classified as (1) methods of measuring objective BE qualities and (2) methods of measuring the perceived environment of urban residents. Absolute (e.g., number, area, width, length, distance), relative (e.g., ratio, density, percent), and composite (e.g., accessibility, connectivity, entropy, which combine more than one measures/indices) measurements were evaluated as objective BE qualities. The attributes of perceived environment were identified from previous related studies [21], and we categorized the perception of the BE into accessibility, aesthetics, safety, convenience, and pleasantness. 

### 2.5. Synthesis of Results 

Vote counting was conducted to summarize the number of studies reporting significant and non-significant findings and the direction of the associations between the BE characteristics and health-related outcomes. The Cochrane handbook indicates that vote counting may be useful when statistical meta-analysis cannot be applied due to the heterogeneity of measured outcomes [22].

In order to clarify the direction of association, we classified the findings as positive (i.e., OR > 1, β > 0) or negative (i.e., OR < 1, β < 0) as a result of the direction of health promotion. The consistency of associations between the BE characteristics and health-related outcomes was determined by five studies or more that reported significant findings to sufficiently indicate consensus [23] (i.e., < 5 studies were classified as “none”). Selected correlates included in ≥5 studies were presented graphically to show n-studies reporting a positive, negative, or non-significant association between the BE characteristics and health-related outcomes. *“Consistent association”* was defined as 75% to 100% of the significant findings reporting the same direction within the BE characteristics (c.f. <75%: *“inconsistent association”*) [23].

## 3. Results

### 3.1. Study Selection

**Figure 1:**
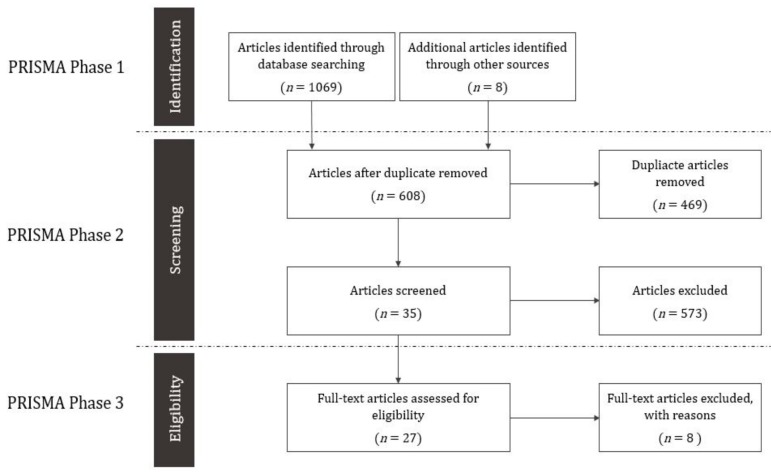
Flow diagram of the study selection.

In the first PRISMA stage, 1077 articles were selected (Figure 1). Of these, 1069 were identified through database searches and eight additional articles were found by searching the reference lists of retrieved studies. In the second stage, 469 duplicate articles were excluded, and two researchers crosschecked the titles and abstracts of the remaining 608 articles. We excluded 573 articles not involving Korean metropolitan cities and/or not analyzing the relationship between the BE and health. In the third stage, we read the full text of the 35 remaining articles and confirmed their eligibility. In the final stage of full text review, 27 articles were selected for analysis. We assigned reference codes for the 27 articles by year of publication and alphabetical order of first author’s name for use in Table 2, Table 3 and Table 4.

### 3.2. Study Characteristics

The number of publications has increased in recent years. Researchers represented 12 fields of study, primarily urban planning and design (39%). Some studies (14%) involved researchers from two disciplines. Only three studies were conducted by public health researchers (11%). Overall, Korean studies tended toward a mono-disciplinary approach from the perspective of urban planning rather than public health.

All studies used a cross-sectional design. The research setting was as follows: 67% in the capital city, 22% in the metropolitan cities, and 11% in both. More than half the studies (56%) had participants of specific age groups, including adolescents (15%), adults (22%), and the elderly (19%), and 44% had participants of all ages. One study [31] used complete enumeration sampling, 59% of the studies used probability sampling, and 37% used non-probability sampling. Primary data were collected via surveys in 52% of the studies, while the rest used secondary data from healthcare, welfare, administration, and culture and sports institutions. About 48% of studies measured objective qualities, 37% measured the perceived environment, and 15% measured both. To examine the relationships among variables, 37% used regression analyses, 33.3% used multi-level analysis or hierarchical linear models to analyze multi-level models, 22.2% used path analysis or structural equation modeling, and two studies used correlational analysis (Table 2).

### 3.3. Health Variables Related to the Built Environment

Health-related variables were classified according to the physical, mental, and social health domain. Most studies (81%) used physical health-related variables to examine the relationship between the BE and health, including health-related behaviors, illness or death, and perceived health status. The health-related behaviors included moderate or high intensity physical activity, walking, sedentary behavior, and dietary behavior, with walking being the most common [29,32,38,40,41,45,46]. Obesity, allergic disease, and mortality were associated with the BE, and several studies reported a correlation between obesity and the BE [26,27,28,35,40,44,48]. Body mass index (BMI) was measured in all studies examining obesity; most studies used the WHO Asia-Pacific regional obesity criteria (BMI ≥ 25). Perceived health status was a variable to identify subjective satisfaction with physical health [33,34,39,49]. The physical health-related variables for physical activity, walking, obesity, and perceived health status had more than four studies.

About one-fifth (19%) of the studies examined the relationship between the BE and mental and social health. The mental health-related variables included prevalence of mental illness and levels of depression, stress, and self-esteem. Of these, depression was used in four studies as a measure of mental health [25,28,30,37]. The social health-related variables were the social activity level, including frequency of social activity, perception of social trust, networks, reciprocity, and social participation among local residents. Only three studies examined the relationship between the BE and social health [25,42,43] and two studies [42,43], in particular, included more social health variables (Table 3).

### 3.4. Associations between the Built Environment Characteristics and Health-Related Outcomes

We investigated the associations between the BE characteristics and health-related outcomes. As noted earlier in Table 1, the BE characteristics were classified as objective or perceived, and the health-related outcomes were grouped into the physical, mental, and social domains (Table 4).

#### 3.4.1. Associations between Land Use and Health-Related Outcomes

**Figure 2:**
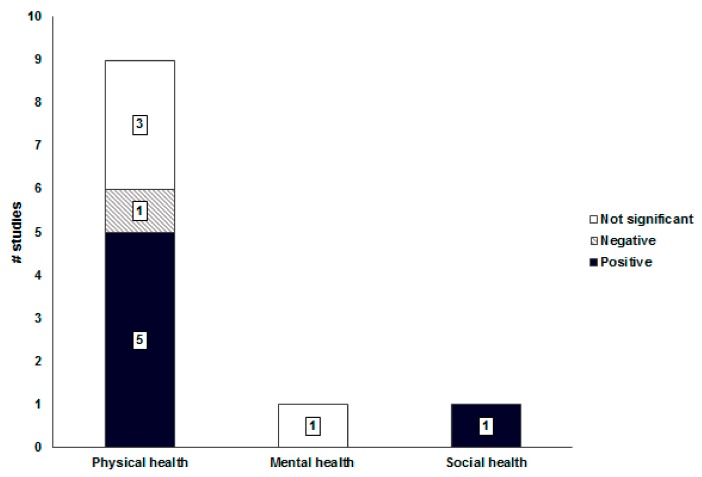
Associations between mixed land use and health-related outcomes.

Mixed land use had an inconsistent association with physical health (Figure 2); five out of nine (56%) studies indicated that mixed land use for residential, commercial, and work purposes had a positive effect on physical activity promotion by inducing walking for the purposes of leisure and travel [28,38,40,46,48]. However, air pollution was higher in areas with high ratios of mixed land use, thus increasing the risk of asthma for residents [47]. Additionally, there was no association between mixed land use and obesity [26,27], and perceived health status [33].

Non-consistent associations were found between mixed land use, mental health, and social health [28,43]. Mixed land use had a non-significant association with depression [28], and only one study [43] reported a positive association between mixed land use and social interaction.

#### 3.4.2. Associations between Street Environment and Health-Related Outcomes

**Figure 3:**
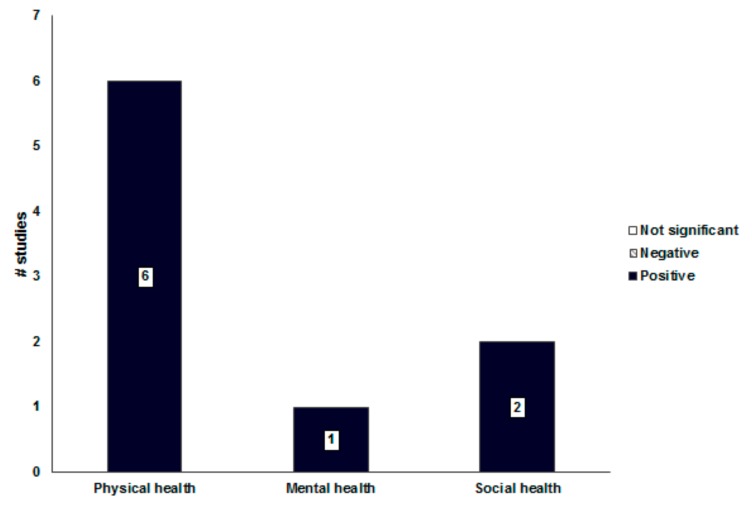
Associations between the street environment and health-related outcomes.

Pedestrian-friendly environments had a positively consistent association with physical health (6/6 = 100%, Figure 3). The higher ratio of pedestrian sidewalk area, crosswalks, and intersections were correlated with the increased walking for exercise and reduced obesity [38,40]. The density of intersections was highly related to the frequency of walking because of the smaller number and lower speed of automobiles and increased street connectivity [38]. The safety, accessibility, pleasantness, and aesthetics of the street environment were correlated with increased walking and perceived health status [32,34,41,49]. Of these, the safety and accessibility of the street environment were the prominent attributes affecting the mobility of vulnerable groups (e.g., older adults and children).

Although positive associations between the street environment and mental health and social health were reported, the consistency of associations was not determined based on only three studies [25,37,43]. The pleasantness of the street environment was correlated with reduced depression and increased social interaction and participation [37,43]. Pleasant streets were an attractive place in the community, providing pedestrian emotional ventilation and opportunities for social interaction.

#### 3.4.3. Associations between Transportation Infrastructure and Health-Related Outcomes

**Figure 4:**
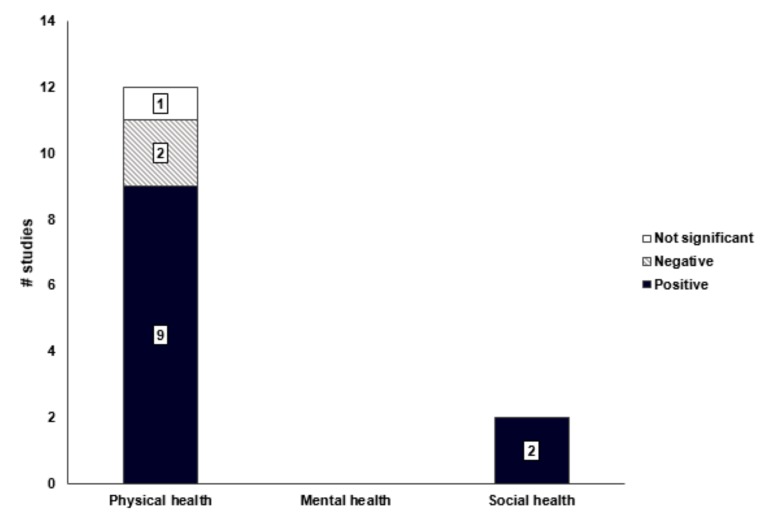
Associations between transportation infrastructure and health-related outcomes.

Transportation infrastructure had a positively consistent association with physical health (9/12 = 75%, Figure 4). The higher density and larger number of public transportation facilities, longer length of bicycle roads, and shorter distance from public transportation facilities were correlated with increased moderate or vigorous physical activity and walking, and reduced obesity [26,27,33,38,40,48,50]. In the current review, the average density of public transportation facilities that induce walking was 19.64/km^2^, and the average distance between them was 0.24 km. However, the larger number of automobile registrations and parking lots, and higher connectivity of roads and speed of vehicles had negative associations on physical health [31,44]. Residents living in urban communities with more vehicles and road connections had higher rates of obesity and lower walking and physical activity frequency.

No studies examined the association between transportation infrastructure and mental health. Although positive associations between public transportation facilities and social health was reported [25,43], the number of such studies did not meet the criteria for determining the consistency of associations. The higher accessibility of public transportation facilities was correlated with increased social interaction and participation [43]. In an urban environment where public transportation was highly accessible, time pressure and psychological burden on social interaction decreased.

#### 3.4.4. Associations of Green and Open Spaces with Health-Related Outcomes

**Figure 5:**
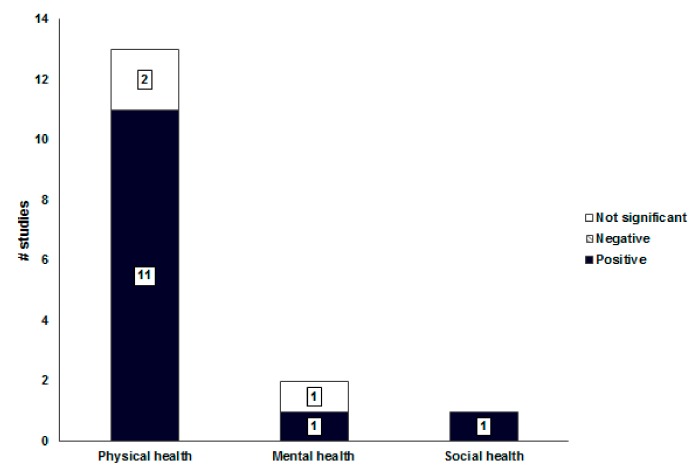
Associations between green and open spaces and health-related outcomes.

Green and open spaces had a positively consistent association with physical health (11/13 = 85%, Figure 5). The higher ratio of parks and green areas in the urban community was correlated with increased physical activity in parks and reduced obesity [26,27,31,36,40,48]. The shorter distance from residences to parks and green areas and higher accessibility to park were correlated with increased physical activity of local residents [28,48,50]. However, the criteria for buffers when measuring the accessibility of parks from residential areas varied across studies. The buffers ranged from 200 to 400 m. Since the distance between neighborhood parks in Korean cities is legally set at 500 m or less (In Korea, the definition of Neighborhood park is a park that is established for the purpose of contributing to health promotion, recreation and emotional life of neighboring residents. Therefore, the distance between neighborhood parks is set by law so that neighboring residents can walk less than 500m and the area is more than 10,000 square meters.), the buffer is generally determined within 500 m. The safety, convenience, accessibility, pleasantness, and aesthetics of green and open spaces were correlated with increased physical activity and walking for leisure [24,41,45].

There were only two studies examining the association between green and open spaces and mental health, and the significance of the findings was inconsistent according to the variables used. Although the area of parks and green spaces was not correlated with depression [30], the shorter distance to parks and green spaces was correlated with reduced stress [40]. Only one study quantified the association between green and open spaces and social health. In the study, the pleasantness of neighborhood parks strengthened the social function as a gathering place for people [42].

#### 3.4.5. Associations between Neighborhood Facilities and Health-Related Outcomes

**Figure 6:**
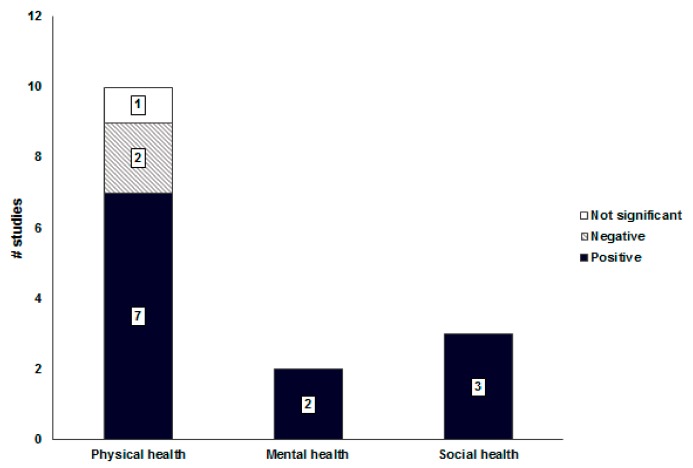
Associations between neighborhood facilities and health-related outcomes.

Neighborhood facilities had an inconsistent association with physical health (7/10 = 70%, Figure 6). The higher density, shorter distance, and larger number of neighborhood facilities, such as welfare centers, schools, restaurants, stores, hospitals, and surveillance, were correlated with increased walking, and reduced obesity and sedentary behaviors [29,40,48,50]. Furthermore, the safety, accessibility, and aesthetics of the neighborhood facilities were correlated with increased moderate or vigorous physical activity, walking, and perceived health status [24,29,39,41,50]. However, the food environment promoted walking for travel but also acted as a risk factor for obesity [26,35]. The larger number of fast food restaurants and convenience stores per unit area was highly correlated with increased obesity of local residents, not only because of physical access but also because of 24-h access.

Neighborhood facilities had positive associations with mental health and social health, but the consistency of associations was not determined (two mental health studies, three social health studies). The higher density and shorter distance of welfare centers were correlated with reduced depression [25]. Perceiving sport facilities and stores as convenient and pleasant had a positive effect on reducing depression [37]. Additionally, the level of social interaction, social trust, and social reciprocity increased the more urban residents perceived their neighborhood facilities as safe and accessible [25,42,43].

## 4. Discussion

This systematic review has clarified the association between the BE and health in compact metropolitan cities. Empirical research on the BE and health in Korean metropolitan scale cities has been conducted primarily in the fields of urban planning and urban design. All studies reviewed were cross-sectional. Among the studies using secondary data, none conducted detailed spatial unit analyses at the community level, which Lee [16] considered a data limitation because the space units did not fit between the secondary environmental data and secondary health data. It is recommended that urban health researchers construct time series data, adjust spatial units of data between public health and urban environmental research, and apply more robust research designs.

The BE characteristics that affected health were land use, street environment, transportation infrastructure, green and open spaces, and neighborhood facilities, which aligns with previous studies [51]. The BE variables were measured in terms of the objective or perceived environments, with the objective variables being more commonly examined. However, recent studies have shown that health behaviors are decisions made through comprehensive evaluations combining both the objective and perceived environment [52,53]. Understanding health behaviors according to the perceived environment has also been emphasized in health promotion strategies [54,55,56]. Using multi-method qualitative research and mixed-methods research can provide greater evidence of the association between the perceived environment and health [21].

The health-related outcomes in the included studies focused on physical health, with few studies including the mental and social health domains. This result supports previous findings suggesting associations between the BE and health lacked an integrated view of health and multi-domain considerations [16]. To overcome this limitation, interdisciplinary discussions and knowledge sharing on the perspectives, concepts, indicators, and measurement methods of urban health research are necessary. Furthermore, international comparative studies may be conducted to consolidate evidence on the health-related variables most affected by urban environmental characteristics. Based on this evidence, an urban health promotion framework can be developed that includes integrated indicators of urban environment characteristics and health-related outcomes.

In previous studies, mixed land use has been reported to be a key strategy for compact city policies that contribute to reduced obesity and promote physical activity [57,58]. However, our findings support that mixed land use has a dual impact on health. Mixed land use increased physical activity and social interaction, but also increased the risk of asthma in children. Some studies have also shown that mixed land use was not associated with obesity and mental health [26,27,28]. This suggests that the effect size and direction of mixed land use on health may be affected by what the types of land use are mixed. There is a need for legal and administrative actions to review and modify land use plans in terms of health promotion.

Pedestrian-friendly environments promote walking and social activities of urban residents. In particular, safety and accessibility were important attributes of the pedestrian-friendly environment [32,34,41,43,49]. Korean metropolitan cities are in the process of transitioning from car-oriented cities to pedestrian-friendly cities, and mixed urban spaces shared by vehicles and pedestrians still remain because of space efficiency [59]. Traffic congestion due to mixed urban spaces was a major risk factor, especially for pedestrian accidents involving children and the elderly. To address these issues, the Korean government has introduced pedestrian zoning and pedestrian-only street design as strategies to create a safe and accessible street environment for pedestrians. However, according to Congiu et al. [60], the urban elements for separation between pedestrian and vehicle areas in a congested traffic environment could interfere with the mutual visibility of pedestrians and vehicles, increasing pedestrian accidents. Urban planning can be established to have a rational street network so that zoning and equipment, which separates the vehicle and pedestrian, do not cause traffic accidents and traffic congestion.

Safety and accessibility can serve as factors for cities to stay active for 24-h, but they can lead to negative health outcomes. Koo et al. [61] pointed out that safe and accessible street environments with high outdoor artificial light at night increase the nighttime activity of urban citizens, which has a negative effect on sleep duration and obesity. This is an urban environment context that has not yet been fully discussed. Urban health research needs to consider analyzing the correlation between health and the street environment.

In our study, transportation infrastructure has been found to be associated with the promotion of physical and social health through public transportation and bicycle use; whereas, automobile use has a negative impact on physical activity and community safety. These findings support the health promotion effect of active transportation that restrains the use of vehicles and promote the use of bicycle and public transportation [62,63]. In addition, we found that the distance of approximately 200 m or the duration of 5- to 10-min on foot between the public transportation and residential areas encouraged walking for travel in a compact urban environment. However, in a study conducted in a non-compact urban environment, public transportation has been recommended to be within a 10- to 15-min walk on foot, or about 400 to 800 m [64]. This suggests that the perceptions of “far” and “close” may be different in the urban form, which can affect walking and patterns of urban mobility.

We found that the proportion of parks and green spaces was associated with physical and mental health. However, if the green area of the city is excessively large, it would interfere with spatial connectivity and have a negative effect on physical, social, and mental health. This supports the hypothesis that open spaces only promote walking up to a certain size threshold [65]. Some studies in the current review have found that the perceived environment of green and open spaces is not directly related to physical health. Green and open spaces are enjoyed as background spaces for residences but may not be used as places of physical activity. Giles-Corti et al. [66] suggested that devising attractive and engaging activities with various purposes for users might help green and open spaces to be community assets for health promotion. When designing green and open spaces, it is necessary to develop spatial awareness and to promote the physical activity of residents by improving the quality of the space and diversifying spatial functions.

When neighborhood living facilities are safe, close and convenient to use, outdoor activities are frequently performed, opportunities for social relationships increase, and depression decreases. In terms of equitable utilization of community assets, physical and economic accessibility and the spatial distribution of neighborhood facilities are related to health equity. This suggests that the strategic utilization of neighborhood facilities as a daily life condition for local residents can lead to the social participation of vulnerable groups and physical health promotion [67]. In our study, the food environment in neighborhood facilities has been reported to have inconsistent associations with health. Although it has promoted walking and social relations, the development of 24-h restaurants and convenience stores has increased the obesity rate in compact metropolitan cities. It is recommended for researchers to consider that BE characteristics might have contradictory effects on health depending on the nature and context of the urban environment.

Overall, we found that BE characteristics in compact metropolitan cities associated to health-related outcomes had accessibility as a common attribute. The accessibility of the BE contributed to health promotion in terms of resource availability. Urban planning and policies for compact cities positively affected walking for travel and physical activity, and promoted social interactions and networks [24,25,26,27,28,29,36,38,39,40,41,48,49,50]. In addition, parks and green areas relieved stress through their utilization and significantly influenced mental health through their close proximity [40].

However, the accessibility of the BE produced some health challenges: 24-h facilities near the residential area increased fatigue and the obesity rate of residents by increasing their nighttime activity and chances of eating later in the evening [26,35]. Mixed traffic streets accessible to both vehicles and pedestrians contributed to an increased risk of conflicts and accidents between pedestrians and motorists [31,44,50], and mixed land use was a risk factor for asthma [47].

The accessibility of the BE is an important health equity issue. Areas with a low density of public transport and neighborhood facilities tended to worsen the physical, mental, and social health of the residents. In urban areas with low access to the BE, physical activity and walking decreased, obesity increased, and residents had difficulty maintaining and forming social relationships [25,42,43]. These health equity issues had a greater impact on vulnerable groups, such as children and the elderly. As the accessibility of the BE decreased, vulnerable groups became less healthy because of the increased cost and effort burdens required to engage in healthy behaviors and social relationships [25,35,37,47,50].

The association between the BE and health studied in the Korean metropolitan cities with a compact urban environment was mostly consistent with that found in urban health research conducted at other urban scales. However, there were differences for some health-related outcomes, the criteria for spatial accessibility, and the BE variable of density. These differences were attributed to the dense and complex urban structure of Korean metropolitan cities and the manner in which the decision-making of health behavior interacts with the perceived environment. Therefore, health promotion strategies aimed at environmental change should consider the environmental specificity and the attributes of the perceived environment related to health behaviors.

Because this study only examined studies published in peer-reviewed journals, high-quality research published outside of peer-reviewed journals was included in the results. This review was limited to studies published from 2011 to 2016, and some studies might have been left out of the review because of the limitations of the search terms, search criteria, and search databases used. Additionally, quality assessment was not conducted, and all the included studies used cross-sectional designs. The causal assumption and true effects are difficult to determine by synthesizing results from cross-sectional studies.

Despite these limitations, due to a systematic review by following PRISMA, the consistency and direction of findings across studies reviewed were rigorously examined. We also identified the BE variables and measurement methods, and health-related variables and measurement methods. These results strengthen the existing evidence for the associations between the BE and health and provide research design and methodological implications for future research on the BE and health.

## 5. Conclusions

This study systematically examined the associations between the BE characteristics and health-related outcomes in compact metropolitan cities in Korea and identified the health benefits and risks of the particular BE characteristics in such cities. All of the reviewed studies were cross-sectional, the study methods and measurement tools varied, and there was insufficient evidence for the associations between the BE characteristics and social and mental health. Future research is called for to focus on the impact of BE characteristics on mental health and social health in a compact urban context, and to use theoretically sound longitudinal designs.

Interdisciplinary urban health strategies are required based on the associations between the BE characteristics, health-related outcomes, and the environmental context. Evidence for the associations between the BE characteristics and health-related outcomes in compact metropolitan cities may provide a greater understanding of the health effects and health behaviors of urban development for compact cities in other countries. A comparative analysis with international studies would contribute to further planning for rational urban development.

## Figures and Tables

**Table 1:** Built environment characteristics and variables from the reviewed articles.

Characteristics	Category	Measurement			
		Objective Qualities of Built Environment			Perceived Environment
		Absolute	Relative	Composite	
Land use	Residential use	Number of households, Number of residents per a room, Residential area	Apartment area ratio, Detached house area ratio, Townhouse area ratio		
	Non-residential use	Industrial area, Industrial floor area, Commercial area, Commercial floor area, Office floor area			
	Mixed land use			Index of mixed land use, Index of residential and non-residential, Index of Population-employees, Entropy index of residential and non-residential, Entropy index of three non-residential use	Accessibility, Convenience
Street environment	Pedestrian sidewalk and pedestrian zone	Pedestrian sidewalk length, width, and area	Pedestrian sidewalk ratio, Ratio of road area to sidewalk area		Safety
	Walking facility and barrier	Number of walking rest facilities, temporary walking barrier, and permanent walking barrier, Number of pedestrian sidewalk lighting facilities			Convenience, Pleasantness
	Intersection	Number of intersections, Number of intersections to population, Number of intersections to employees	Density of intersections, 4-way intersection ratio		Accessibility, Safety
	Crosswalk	Number of crosswalk subsidiaries, Number of traffic lights	Crosswalk density		Accessibility Safety
	Street connectivity and hierarchy	Number of sidewalk cuts		Entropy index of street hierarchy	Accessibility
	Building and block	Building height, Block size	Window ratio of first floors		Aesthetics, Pleasantness
Transportation infrastructure	Bicycle road	Length of bicycle roads		Bicycle road accessibility	Accessibility
	Bus stop and route	Number of bus stops, Number of bus routes, Bus stop distance	Bus stop density	Bus stop accessibility	Accessibility, Convenience
	Subway and railway	Number of subway stations, Railway station distance	Subway station density	Subway accessibility	Accessibility, Convenience
	Vehicle use	Number of car accidents, Passing vehicle speed			Safety
	Parking	Number of parking lots, Number of illegally parked cars			
	Road	Number of road lanes	Road density	Road connectivity	Safety
Green and open spaces	Park	Number of parks, Number of park entrances, Total area of parks, Park shortest network distance, Park shortest straight distance	Park area ratio, Ratio of park area to city area, Park area per capita	Park accessibility	Accessibility, Aesthetics, Convenience, Pleasantness
	Green spaces	Total area of green spaces	Green spaces area ratio, Green spaces per capita		Accessibility, Pleasantness
	Open spaces		Open spaces area ratio		Safety
Neighborhood facilities	Food environment	Number of traditional markets, Number of large-scale marts, Number of groceries, Number of street vendors, Number of fast food restaurants, Number of snack bars, Number of convenient stores	Fast food restaurants per area, Convenient stores per area	Accessibility to large-scale marts, Accessibility to traditional market	Accessibility, Convenience
	Healthcare facility	Number of medical facilities		Accessibility to medical facility	Accessibility, Convenience
	Education facility	Number of schools	Schools per area		Accessibility, Convenience, Pleasantness
	Community facility	Number of welfare centers, Number of sports facilities		Accessibility to elderly welfare center, Accessibility to sports facility, Index of mixed community facilities	Accessibility, Convenience
	Retail shop	Number of stores		Store accessibility	Accessibility, Convenience
	Surveillance	Number of CCTV			Safety

**Table 2:** Characteristics of the reviewed articles.

Authors (year)	Research Fields	Setting	Participants (Age)	Sample Size	Sampling a	Data b	BE Measurement	Statistical Analysis
Kang and Kim (2011) [24]	Sport sciences	C	Older adults (65+)	290	Non-probability	1	O, P	MR
Kim and Ahn (2011) [25]	Civil and environmental engineering	C	Older adults (60+)	381	Non-probability	1	O	SEM
Kim and Kang (2011a) [26]	Urban planning	C	Residents (All ages)	NR	Probability	2	O	SR
Kim and Kang (2011b) [27]	Urban planning	C	Residents (All ages)	1982	Probability	2	O	MA
Sung (2011) [28]	Transportation	C	Adults (19+)	976	Probability	2	O	MA
Kim et al. (2012) [29]	Sport sciences	C	Older adults (65+)	418	Probability	1	O, P	LR
Ko and Lee (2012) [30]	Social welfare	C	Older adults (65+)	1413	Probability	2	O	HLM
Lee and Joo (2012) [31]	Urban and regional planning	C	Residents (All ages)	NR	Other	2	O	SR
Lee and Shepley (2012) [32]	Landscape architecture	C	Residents (All ages)	412	Non-probability	1	P	PA
Choi and Kim (2013) [33]	Urban planning and engineering	M	Residents (All ages)	1329	Probability	2	O	HLM
Kim and Kim (2013) [34]	Social welfare	C	Residents (All ages)	45,605	Probability	2	P	MA
Park et al. (2013) [35]	Medicine/Public health	C	Adolescents (11–16)	939	Probability	1	O	MA
Park et al. (2013) [36]	Medicine	C, M	Residents (All ages)	4,055	Probability	2	O	MR
Lee and Choi (2014) [37]	Housing environmental design	C	Adolescents (17)	446	Non-probability	1	P	MR
Sung et al. (2014) [38]	Transportation/Urban planning and engineering	C	Residents (All ages)	1823	Probability	1	O	MA
Jung and Lee (2015) [39]	Urban planning	C, M	Older adults (65+)	11,407	Probability	2	P	SEM
Kim and Kim (2015) [40]	Urban planning	M	Adults (19+)	NR	Probability	2	O, P	CA
Lee et al. (2015) [41]	Landscape architecture	M	Residents (All ages)	303	Non-probability	1	P	LR
Yoo and Lee (2015) [42]	Urban planning and engineering	M	Residents (All ages)	9,406	Probability	2	P	SEM
Cho and Lee (2016) [43]	Urban planning and engineering	C	Adults (19–64)	484	Non-probability	1	P	SEM
Chun (2016) [44]	Urban and regional planning	C	Adults (19–64)	NR	Probability	2	O	SR
Jang et al. (2016) [45]	Landscape architecture	M	Residents (All ages)	143	Non-probability	1	P	MR
Kim et al. (2016) [46]	Sport science	C	Adults (20–59)	1407	Probability	1	P	CA
Kim et al. (2016) [47]	Public health/Environmental science	C, M	Adolescents (9–13)	4404	Non-probability	1	O	LR
Lee and Lee (2016) [48]	Urban planning	C	Adults (19+)	5692	Probability	2	O	MA
Lee et al. (2016) [49]	Landscape architecture	M	Residents (All ages)	278	Non-probability	1	P	PA
Lee et al. (2016) [50]	Public health/Food science and nutrition	C	Adolescents (12–13)	1134	Non-probability	1	O, P	HLM

Notes: C: capital city; CA: correlation analysis; HLM: hierarchical linear model; LR: logistic regression; M: metropolitan cities; MA: multilevel analysis; MR = multiple regression; NR: the contents were not reported in the study; O: objective qualities of built environment; P: perceived environment; PA: path analysis; SEM: structural equation model; SR: spatial regression. ^a^ Sampling classification: Non-probability = convenience sampling, purposive sampling, and quota sampling; Other = complete enumeration sampling; Probability = random sampling, cluster sampling, stratified sampling, and systematic sampling. ^b^ Data classification: 1 = primary data; 2 = secondary data.

**Table 3:** Health-related variables from the reviewed articles.

Health Domain	Variables		Measurement	
			Objective	Subjective
Physical health	Health-related behaviors	Eating behaviors	[35]	
		Moderate or vigorous physical activity	[36,40,48,50]	[24]
		Sedentary behaviors	[50]	
		Walking ^a^	[29,32,38,40,41,45,46]	[32]
	Illness or Death	Allergic diseases	[47]	
		Mortality rate	[31]	
		Obesity	[26,27,35,40,44,48]	[28]
	Perceived health status			[33,34,39,49]
Mental health	Depression			[25,28,30,37]
	Self-efficacy			[37]
	Stress			[28,40]
Social health	Social interaction		[25]	[42,43]
	Social participation			[43]
	Social reciprocity			[42,43]
	Social trust			[42,43]

^a^ Indicator includes walking for the purposes of recreation and travel.

**Table 4:** Associations between the built environment characteristics and health-related outcomes.

Characteristics of Built Environment	Association with Health Promotion	Objective Qualities of Environment			Perceived Environment				
		Absolute (number, area, width, length, distance)	Relative (ratio, density, percent)	Composite (combined index)	Accessibility	Aesthetics	Convenience	Pleasantness	Safety
Land use	Positive	[48] ^a,1^	[48] ^a,1^	[28] ^a,2^, [38] ^a,1^, [40] ^a,2^	[43] ^c,2^, [46] ^a,1^		[46] ^a,1^		
	Negative	[47] ^a,1^							
	Null		[33] ^a,2^	[26] ^a,1^, [27] ^a,1^, [28] ^b,2^, [33] ^a,2^					
Street environment	Positive	[25] ^c,1^, [38] ^a,1^	[38] ^a,1^, [40] ^a,1^	[38] ^a,1^	[34] ^a,2^, [41] ^a,1^, [43] ^c,2^, [49] ^a,2^	[49] ^a,2^		[37] ^b,2^, [43] ^c,2^, [49] ^a,2^	[32] ^a,1^, [34] ^a,2^, [41] ^a,1^, [49] ^a,2^
	Negative								
	Null								
Transportation infrastructure	Positive	[25] ^c,1^, [33] ^a,2^, [38] ^a,1^, [40] ^a,2^, [48] ^a,1^	[26] ^a,1^, [27] ^a,1^, [40] ^a,2^	[25] ^c,1^	[24] ^a,2^, [39] ^a,2^, [43] ^c,2^		[40] ^a,2^		
	Negative	[44] ^a,1^	[31] ^a,1^						
	Null	[33] ^a,2^							
Green and open spaces	Positive	[40] ^b,2^, [48] ^a,1^, [50] ^a,1^	[26] ^a,1^, [27] ^a,1^, [31] ^a,1^, [36] ^a,1^, [40] ^a,1^, [48] ^a,1^	[28] ^a,2^	[24] ^a,2^, [41] ^a,1^, [45] ^a,1^		[45] ^a,1^	[41] ^a,1^, [42] ^c,2^, [45] ^a,1^	[45] ^a,1^
	Negative								
	Null		[30] ^b,2^, [33] ^a,2^, [44] ^a,1^	[33] ^a,2^					
Neighborhood Facility	Positive	[25] ^b,2^, [29] ^a,1^, [40] ^a,1^, [50] ^a,1^	[40] ^a,1^, [48] ^a,1^	[25] ^b,2, c,1^	[24] ^a,2^, [29] ^a,1^, [39] ^a,2^, [41] ^a,2^, [43] ^c,2^	[41] ^a,1^	[37] ^b,2^	[37] ^b,2^	[42] ^c,2^, [43] ^c,2^, [50] ^a,1^
	Negative		[26] ^a,1^, [35] ^a,1^						
	Null		[27] ^a,1^						

^a^ Article related to physical health; ^b^ Article related to mental health; ^c^ Article related to social health; ^1^ Objective health-related measure; ^2^ Subjective health-related measure.
